# Simple in-line stopped flow photolysis of copper complexes in natural waters using a flow injection system

**DOI:** 10.1155/S1463924692000026

**Published:** 1992

**Authors:** Michael H. I. Comber, Gordon J. Eales, Peter J. D. Nicholson, Simon P. Henn

**Affiliations:** 1 ICI Group Environmental Laboratory Freshwater Quarry Devon Brixham TQ5 8BA UK; 2 Department of Chemistry and Physics Nottingham Polytechnic Clifton Lane Nottingham NG11 8NS UK

## Abstract

The development of an in-line digestion system based on simple UV lamp is reported. The effect of photolysis on the preconcentration of copper was investigated using an in-line Chelex-l00 ion-exchange column linked to an atomic absorption spectrometer. Three model ligands, glycine, NTA and EDTA, have been used to demonstrate the effect of the digestion system. In a stopped-flow mode, over 90% of the complexed copper was recovered in the presence of any of the three ligands. When the UV lamp was turned off, this changed to 84, 45 and 2% recovery for the glycine, NTA and EDTA complexed copper, respectively. The ability to analyse samples with the UV lamp on or off means that the device may be used to study the speciation of the copper.


Journal of Automatic Chemistry, Vol. 14, No. 1 (January–February 1992), pp. 5–8

### Simple in-line stopped flow photolysis of copper complexes in natural waters using a flow injection system

**Michael H. I. Comber, Gordon J. Eales, Peter J. D. Nicholson**
*ICI Group Environmental Laboratory, Freshwater Quarry, Brixham, Devon TQ5 8BA, UK*

**and Simon P. Henn**
*Department of Chemistry and Physics, Nottingham Polytechnic, Clifton Lane, Nottingham NG11 8NS, UK*

*The development of an in-line digestion system based on simple UV lamp is reported. The effect of photolysis on the preconcentration of copper was investigated using an in-line Chelex-100 ion-exchange column linked to an atomic absorption spectrometer. Three model ligands, glycine, NTA and EDTA, have been used to demonstrate the effect of the digestion system. In a stopped-flow mode, over 90% of the complexed copper was recovered in the presence of any of the three ligands. When the UV lamp was turned off, this changed to 84, 45 and 2% recovery for the glycine, NTA and EDTA complexed copper, respectively. The ability to analyse samples with the UV lamp on or off means that the device may be used to study the speciation of the copper.*

When determining metal ions in natural waters by atomic spectrometry, it is frequently necessary to use a preconcentration technique due to the insensitivity of most instruments. Those instruments that are capable of determining metals directly, for example inductively coupled plasma mass spectrometry or graphite furnace atomic absorption spectrometry, suffer from severe matrix effects, and require a separation of the ions of interest from the matrix. Early use of the Chelex-100 resin [1] demonstrated its usefulness in the pre-concentration and determination of metals in seawater. Subsequent studies showed that, while complexation reduced the recovery, appropriate choice of conditions led to the development of speciation procedures [2–4].

The advantages of flow injection analysis/atomic absorption spectrometry (FIA/AAS) have been demonstrated in a number of previous studies. These include a reduced sample size, increased speed and sensitivity and improved reproducibility [5–7]. The conditions necessary to obtain these improvements have also been reported. The advantages of carrying out the pre-concentration in-line include reduction of the sample volume from typically 1000 to less than 10 ml; a reduction in the time to carry out the determination; and an increased capacity for automation.

The need for pre-treatment of environmental water samples in order to obtain complete recovery of dissolved metals is due to the complexation of the metals by ligands. This is well known, and systems to facilitate such treatment, have included UV photolysis [9], and heat digestion [9, 10]. Putting such treatment in-line would have all the advantages described above, as well as the ability to discriminate between complexed and uncomplexed metal ions. Despite these advantages, however, the use of an in-line digestion system has not been reported for the determination of total dissolved metals in water. Previously reported in-line digestion systems have been aimed at the total destruction of organic compounds for the quantification of organic carbon. Van Steenderen *et al.* [11,12], for example, reported a system using UV with persulphate; they noted that approximately 50% of added EDTA was destroyed by UV alone. Another system used titanium dioxide absorbed on a Teflon tube wound round a UV lamp [13]. Finally a system used for environmental samples has been described, in which the use of an in-line digester was used for the determination and speciation of phosphorus in fresh water [14].

The system described in this paper is based on the very simple UV lamp found in a RS EPROM eraser, around which is wound Teflon tubing, through which the sample travels. Apart from this, the system is similar to several previously described [5–8]. The sample is injected into the carrier stream, and, when in the digester, the flow is stopped for a set period so that the metal complexes can be destroyed. The flow is then restarted and the metal pre-concentrated on the Chelex-100 column, prior to determination by AAS.

The choice of ligands to simulate those found in the environment was based on previous experience, and the fact that they will cover the range of naturally occurring ligands. The ability of the model ligands to complex copper was confirmed by Martell *et al.* [15], who demonstrated using the modelling program MINEQL, that in seawater copper would be predominantly complexed by EDTA or NTA at concentrations as low as $10^{-9}$ M.

### Experimental

### *Reagents and apparatus*

Deionized water was used throughout, and all chemicals were analytical reagent grade. Standard solutions of copper were prepared by suitable dilutions of a 1000 mg $l^{-1}$ solution in 1 M nitric acid (Fisons plc, Scientific Equipment Division, Bishop Meadow Road, Loughborough, Leicestershire LE11 0RG, UK). All reagents were stored in clean polythene bottles.

The ammonium acetate buffer was obtained by the dilution of a 2 M stock, prepared by mixing 55·5 ml of 99% acetic acid with 112·5 ml of 25% ammonia solution, diluted to 500 ml with deionized water.

© Copyright ICI Group 1992

2 M nitric acid was prepared by the dilution of Analar grade nitric acid, s.g. 1·42.

Chelex-100, 50–100 mesh, sodium form (Bio-Red Laboratories Ltd, Bio-Rad House, Marylands Avenue, Hemel Hempstead, Hertfordshire HP2 7TD, UK), was used as received.

The FI system comprised of a Watson-Marlow peristaltic pump (Watson Marlow Ltd, Falmouth, Cornwall TF11 4RU, UK); Dionex pneumatically operated Teflon valves (Dionex [UK] Ltd, 4 Albany Corut, Camberley, Surrey GU15 2PL, UK); and standard 2 mm i.d. Teflon tubing. The preconcentration column was prepared from a glass microcolumn, 3 mm i.d., 50 mm long (Anachem Ltd, Charles Street, Luton, Bedfordshire LU2 0EB, UK), packed with the Chelex-100, converted to the ammonia form.

The FI system was built according to details described previously [5–8], with a water boost line to the AAS, and similar operating parameters. The flow rate to the AAS was set at 6 ml min$^{-1}$, and an injection volume of 1 ml used. The effluent from the UV digester was buffered by the addition of ammonium acetate, prior to entering the Chelex column.

The UV digester was made by drilling two small holes in the side of an RS 424-254 EPROM eraser (RS Components Ltd, PO Box 99, Corby, Northamptonshire NN17 9RS, UK). The Teflon tubing was then fed into the EPROM box, and 3 m of tubing wound tightly around the 8 W UV lamp.

The atomic absorption spectrometer was a Perkin Elmer 2380 (Perkin Elmer Ltd, Maxwell Road, Beaconsfield, Buckinghamshire HP9 1QA, UK), fitted with an air-acetylene burner. All instrument parameters were as recommended by the manufacturer.

Recordings were made on a Hewlett-Packard 3390A integrator (Hewlett-Packard Instruments Ltd, King Street Lane, Winnersh, Wokingham, Berkshire RG11 5AR, UK); and a JJ Instruments PLF record (JJ Instruments Ltd, Brook Avenue, Warsash, Southampton SO3 6HP, UK).

Direct analysis of off-line extracted samples, and direct determination of copper was determined using an ARL3510 Inductively Coupled Plasma Optical Emission Spectrometer, ICPOES (Fisons Instruments, Sussex Manor Park, Gatwick Road, Crawley, Sussex RH10 2QQ, UK). The operating conditions were as recommended by the instrument manufacturer. The copper was measured at an emission line of 324·754 nm.

*Procedure*

In the load position (see figure 1[*a*]), the sample loop is flushed with the sample, while the Chelex column is washed with 2 M nitric acid. In this position the previous

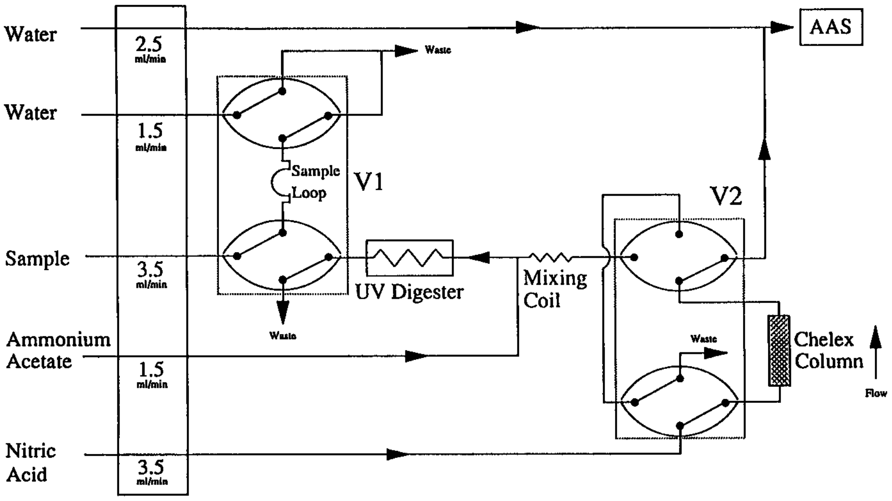

*Figure 1(a). LOAD position – sample being flushed through loop, and Chelex column being flushed with nitric acid.*

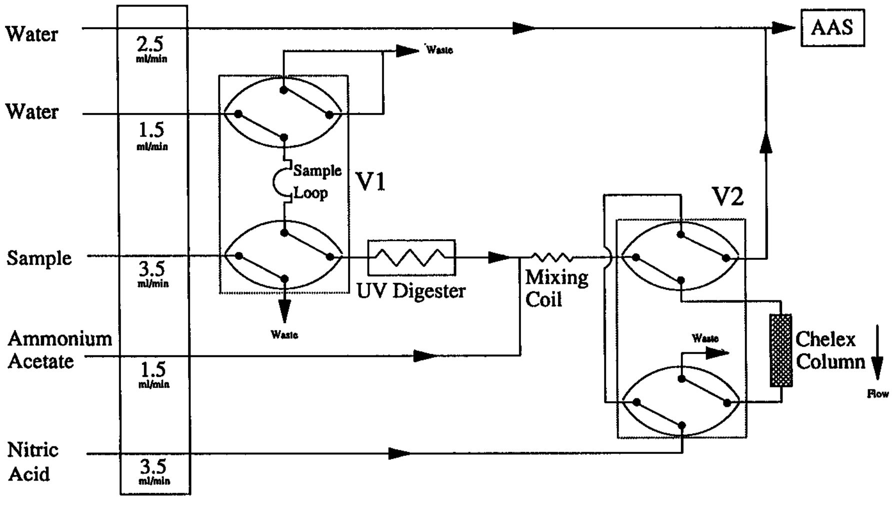

*Figure 1(b). INJECT position – sample injected into UV digester, and onto the Chelex column.*

*Table 1. Effect of changing ligand concentration on the recovery of copper by in-line Chelex extraction.*

| | Concentration of copper: 1 mg/l | | |
|---|---|---|---|
| Concentration of ligand (mg/l) | % recovery Glycine | NTA | EDTA |
| 1 | 100 | 73 | 76 |
| 3 | 100 | 25 | 58 |
| 10 | 100 | 25 | 16 |
| 20 | 100 | 24 | 15 |

Each recovery was the mean of three separate determinations, the standard deviation was always less than 5%.

sample is being analysed, as any copper present on the Chelex column is washed off, and into the AAS. Operation of the valves to the inject position (see figure 1[*b*]), then injects the sample into the carrier stream and it passes to the Chelex column via the UV digester. The time a sample took to reach the coil in the UV digester was determined visually by injecting dye. In the stopped flow mode this was then the time at which the flow was stopped.

Total analysis time using this procedure in a flow through mode was less than 4 min.

When not in use, the Chelex column was stored in its regenerated form ready for use, and water pumped through the system, excluding the Chelex column, to wash out any reagents, and to prevent back flow into the reagent bottles.

### Results

### *Effect of ligands*

The effect of adding varying concentrations of glycine, NTA and EDTA, was investigated (see table 1), and the decline in recovery with increased concentration of EDTA and NTA is shown. This demonstrated that, at a ligand concentration of 10 mg/l, the amount of copper being recovered was minimized. All the subsequent recovery experiments were then carried out at a concentration of 10 mg/l of the ligand.

The recovery of copper when the UV lamp was turned on, and the system operated in the stopped flow mode was then investigated. The results (see figure 2) clearly demonstrate that the copper was easily released from weak complexes, as demonstrated by glycine, but that a 10 min digestion period was required for full recovery of the copper-EDTA complexes. The recovery of copper from this in-line system is similar to that produced by an off-line system.

The FIA-AAS system described is thus capable of being used to analyse natural water samples in one of two modes:

(1) Labile copper – UV digester off, pre-concentration onto the Chelex column, followed by elution with nitric acid and determination of copper by AAS.

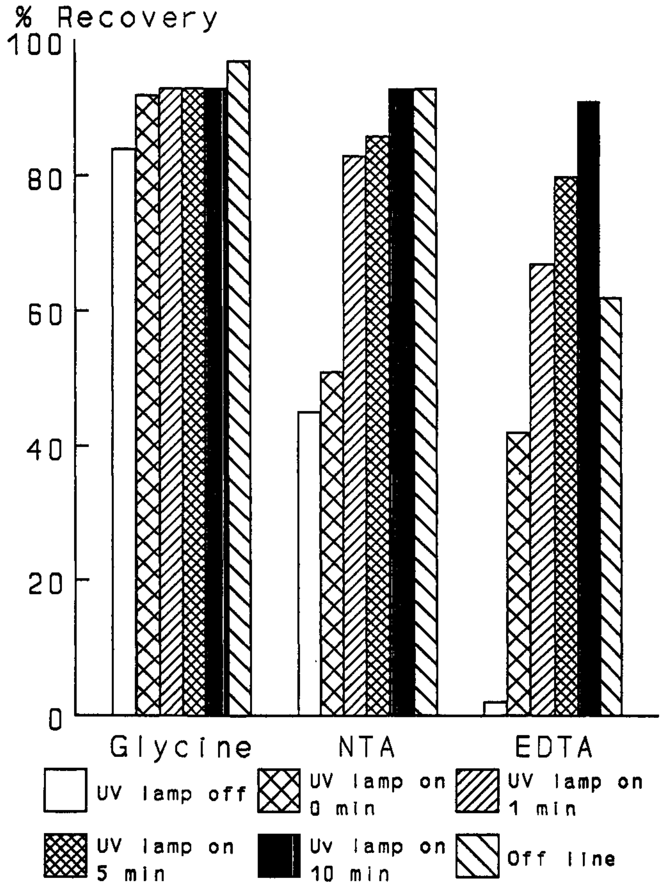

*Figure 2. Recovery of copper by Chelex extraction, comparison of off-line extraction to in-line extraction with and without in-line digestion.*

(2) Total copper – UV digester on, stop flow for 10 min, followed by pre-concentration onto the Chelex column, elution with nitric acid and determination of copper by AAS.

The system described was set up to demonstrate the feasibility of the in-line digester. With the present system the detection limits are too high to allow for comparison of natural waters. However, work is in progress to link this system to more sensitive instruments, thus allowing for this comparison.

The ICI Group Environmental Laboratory takes part in the Aquacheck scheme, for inter-laboratory comparison of analyses of natural waters, organised by the Water Research Centre, at WRc Environment, Medmenham Laboratory, Henley Road, Medmenham, PO Box 16, Marlow, Buckinghamshire SL7 2HD, UK. A sample sent to the laboratory recently was analysed by the FIA-AAS system, with the digester on, and a value for copper of 71 µg/l obtained. This compares with a certified value of 69 µg/l.

### Conclusions

The advantages of FIA-preconcentration-AAS have been frequently demonstrated, and include improved sensitivity, faster analyses and reduced matrix effects. The system described demonstrates a further advantage of being able to carry out in-line sample treatment, thus allowing for the determination of total copper. By substituting a more sensitive spectrometer into the system, speciation determinations may be performed, by simply having the UV lamp on or off.

### Acknowledgements

One of the authors, S. P. H. would like to thank ICI plc, for supporting him in this work. The useful comments made by Dr D. Taylor are acknowledged. The authors wish to thank ICI plc for permission to publish this paper.

### References

1. Riley, J. P. and Taylor, D., *Analytica Chimica Acta,* **40** (1968), 479.
2. Figura, P. and McDuffie, B., *Analytical Chemistry,* **52** (1980), 1433.
3. Florence, T. M., *Analytica Chimica Acta,* **141** (1982), 73.
4. Shibu, M. P., Balchand, A. N. and Nambisan, P. N. K., *Science of the Total Environment,* **97/98** (1980), 267.
5. Olsen, S., Pessanda, L. C. R., Ruzicka, J. and Hansen, E. H., *Analyst,* **108** (1983), 905.
6. Fang, Z., Xu, S. and Zhang, S., *Analytica Chimica Acta,* **200** (1987), 35.
7. Hartenstein, S. D., Ruzicka, J. and Christian, G. D., *Analytical Chemistry,* **57** (1985), 21.
8. Brown, M. W. and Ruzicka, J. *Analyst,* **109** (1984), 1091.
9. Florence, T. M. and Batley, G. E., *Talanta,* **23** (1976), 179.
10. Abdullah, M. I., El-Rayis, O. A. and Riley, J. P., *Analytica Chimica Acta,* **84** (1976), 363.
11. van Steenderen, R. A., Basson, W. D. and van Duuren, F. A., *Water Research,* **13** (1979), 539.
12. van Steenderen, R. A. and Lin, J.-S., *Analytical Chemistry,* **53** (1981), 2157.
13. Low, G. K.-C. and Mathews, R. W., *Analytica Chimica Acta,* **231** (1990), 13.
14. McKelvie, I. D., Hart, B. T., Cardwell, T. J. and Cattrall, R. W., *Analyst,* **114** (1989), 1459.
15. Motekaitis, R. J. and Martell, A. E., *Marine Chemsitry,* **21** (1987), 101.

